# ISO/TS 21564:2019- based Evaluation of a Semantic Map between Variables in the ISARIC Freestanding Follow Up Survey and ORCHESTRA Studies

**DOI:** 10.1007/s10916-023-02012-4

**Published:** 2023-11-14

**Authors:** Eugenia Rinaldi, Sylvia Thun, Caroline Stellmach

**Affiliations:** Core Facility Digital Medicine and Interoperability, Berlin Institute of Health (BIH) at Charité - Universitätsmedizin Berlin, Anna-Louisa-Karsch-Str.2, 10178 Berlin, Germany

**Keywords:** ISARIC, Clinical study data, Case report form, Interoperability, Infectious diseases, Semantic standards, COVID-19, ISO/TS 21564:2019, Data sharing

## Abstract

The COVID-19 pandemic has led to tremendous investment in clinical studies to generate much-needed knowledge on the prevention, diagnosis, treatment and long-term effects of the disease. Case report forms, comprised of questions and answers (variables), are commonly used to collect data in clinical trials. Maximizing the value of study data depends on data quality and on the ability to easily pool and share data from several sources. ISARIC, in collaboration with the WHO, has created a case report form that is available for use by the scientific community to collect COVID-19 trial data. One of such research initiatives collecting and analyzing multi-country and multi-cohort COVID-19 study data is the Horizon 2020 project ORCHESTRA. Following the ISO/TS 21564:2019 standard, a mapping between five ORCHESTRA studies’ variables and the ISARIC Freestanding Follow-Up Survey elements was created. Measures of correspondence of shared semantic domain of 0 (perfect match), 1 (fully inclusive match), 2 (partial match), 4 (transformation required) or 4* (not present in ORCHESTRA) as compared to the target code system, ORCHESTRA study variables, were assigned to each of the elements in the ISARIC FUP case report form (CRF) which was considered the source code system. Of the ISARIC FUP CRF’s variables, around 34% were found to show an exact match with corresponding variables in ORCHESTRA studies and about 33% showed a non-inclusive overlap. Matching variables provided information on patient demographics, COVID-19 testing, hospital admission and symptoms. More in-depth details are covered in ORCHESTRA variables with regards to treatment and comorbidities. ORCHESTRA’s Long-Term Sequelae and Fragile population studies’ CRFs include 32 and 27 variables respectively which were evaluated as a perfect match to variables in the ISARIC FUP CRF. Our study serves as an example of the kind of maps between case report form variables from different research projects needed to link ongoing COVID-19 research efforts and facilitate collaboration and data sharing. To enable data aggregation across two data systems, the information they contain needs to be connected through a map to determine compatibility and transformation needs. Combining data from various clinical studies can increase the power of analytical insights.

**Supplementary information:**

The online version contains supplementary material available at 10.1007/s10916-023-02012-4.

## Background

The worldwide socio-economic and political impacts of the COVID-19 pandemic have led to a rapid rise in COVID-19-focused clinical studies in order to collect data on the SARS CoV-2 infection. These studies cover a large cross-section of information relevant to viral infections, including diagnosis, treatment (including vaccinations), socioeconomic impact and many more [1, 2].

Large public and private institutions have been funding research consortia and clinical studies with the expectation of increasing the body of research and knowledge on the SARS-CoV-2 virus [3].

One of the COVID-19-focused, European Commission funded research projects is ORCHESTRA. Starting in 2020, partners have been working on creating a pan-European cohort of COVID-19 patients to advance knowledge on the disease [4].

The key component of any study is the information gained from enrolled patients following the study protocol. This data is used for analysis to answer the defined research question. It is common to use either paper-based or electronic case report forms (CRFs) [5–8] to record patient information, at one or more time points, following a visit schedule. A CRF is made of variables that represent questions and their respective answers; answers can either be free text or they are coded, presenting choices from a pre-defined list. A CRF must be meticulously designed to enable efficient collection of relevant, high-quality data and can therefore be very resource consuming [9].

To relieve the study-startup effort and in an attempt to standardize data collection, CRFs for COVID-19 data collection have been created and made available to the public by the International Severe Acute Respiratory and Emerging Infection Consortium (ISARIC) in collaboration with the World Health Organization (WHO) [10]. ISARIC’s first COVID-19 CRF was published at the end of January in 2020 and is an adapted version of the Clinical Characterisation Protocol (CCP) which had been created in 2012 to support harmonized data collection during disease outbreaks [11].

Similarly, the German Corona Consensus (GECCO) data set definition was developed as a data model that builds on semantic and syntactic interoperability standards for the collection of COVID-19 data [12]. The ORCHESTRA project adapted these prior efforts to standardize data collection for its new, ongoing and already completed clinical studies. ORCHESTRA studies have a wide range of informational depth and focus areas; including fragile population, healthcare-workers, serology parameters, vaccination monitoring and long-time sequelae (“Long-COVID”) [13] tracking.

All variables (questions and answers) that are part of clinical studies in ORCHESTRA were harmonized and mapped to international terminology standards. Additionally, common data elements between the studies were identified [14]. Semantic standards, such as ontologies and classifications (i.e., the WHO’s International Classification of Diseases) assign standard codes to clinical concepts to unambiguously identify them. Syntax standards provide a uniform exchange format to health data [15]. Together, these standards foster interoperability of health data, the ability to exchange information between systems, and to read and use the received information [16].

A key benefit of uniting multiple partners and clinical studies within one research consortium is the ability to perform shared analysis. Depending on applicable national data protection regulations and informed consent forms, this analysis could be done on the patient data directly or through federated analysis [17] of anonymized, de-identified data. The prerequisite for any shared analysis is the availability of harmonized data. When comparing two datasets, harmonization often requires creating a map between the two to identify data elements that represent the same concepts [18]. The ISO/TS 21564:2019 Health Informatics, Terminology resource map quality measure [19] provides a standard for evaluating such a map between two datasets.

Given the importance of sharing data in the field of COVID-19 research, we have performed a semantic mapping between ORCHESTRA study variables and those defined in the ISARIC Freestanding Follow Up Survey CRF [20] (herein after referred to as ISARIC FUP CRF). This map provides insights into the variables for which joint analysis with the ORCHESTRA project would be possible. Any other study having adopted the ISARIC FUP CRF for data collection would, taking into account applicable data protection legislations, be theoretically able to share data and perform joint analysis with ORCHESTRA on their data for these variables. The performed mapping between ORCHESTRA study and ISARIC FUP CRF variables was evaluated using the ISO/TS 21564:2019 Health Informatics, Terminology resource map quality measures.

## Methods

Authors jointly created a map to investigate the measure of shared semantic domain between variables used in the ISARIC Freestanding Follow Up Survey (V1.1 10 March 2021), and the pool of defined CRF variables used across four project work packages and six observational studies in ORCHESTRA. The ORCHESTRA variables considered belong to the following studies:

- “Follow-up of Covid-19 Long Term Sequelae“ study (NCT05097677) [21]
- “Monitoring COVID-19 Vaccination Response in Fragile Populations (ORCHESTRA-4)” (NCT05222139) [22]
- KoCo19 (prospective Covid-19 cohort Munich) study [23]
- KoCoImpf study [24]
- Umbrella CRF of healthcare worker clinical studies

The quality of the map for each variable was annotated directly in the ISARIC CRF file and documented as a complete map in an Excel file. The mapping process followed a consensus approach between the study authors.

The quality and utility of the map was assessed following the methodology described in the ISO/TS 21564:2019 Health Informatics, Terminology resource map quality measures (MapQual). The ISARIC FUP CRF variables were regarded as source code system and, for each variable, we searched the pool of six ORCHESTRA clinical studies’ variables, which were considered the target code system to determine the level of matching. Following the ISO specifications, we used determinant 2 to evaluate the mapping within a shared semantic domain. A score of 0 was assigned to variables that were a perfect match, meaning the same question with its respective answers defined in the ISARIC FUP CRF was also used in one or more ORCHESTRA studies. A score of 1 was noted for ISARIC variables that were also present in ORCHESTRA studies but for which the ISARIC FUP CRF collected more information than covered in the ORCHESTRA studies. The score of 2 denoted variables that were a partial match, meaning each code system covered part of the other but neither encompassed the entire meaning of the variable. A score of 4 was assigned if an ISARIC FUP CRF variable would require a complex transformation to find a match in ORCHESTRA. In our approach, we considered also the efficiency with which the concept captured in a variable could be transferred from one CRF to the other. When the transformation involved analysis of more than two variables in ORCHESTRA to obtain the corresponding ISARIC FUP CRF information, we assigned a score of 4. Although not included in the ISO standard, we added an additional score of 4* to mark variables that were entirely absent in ORCHESTRA studies’ variables. In case that several target studies in ORCHESTRA were found to show a match to a source variable, a score was assigned only for the best match. However, the mapping table (Supplementary Table 1) reflects all matching variables.

## Results

In our effort to create a map between the ISARIC FUP CRF and the data captured in six ORCHESTRA study CRFs, we identified 160 variables (including sub questions) that comprise the ISARIC FUP CRF. Eight of these variables were dates (format: DD/MM/YYYY), 16 were descriptive headers, 18 variables required free text answers and 118 variables provided fixed answer choices.

**Fig. 1:**
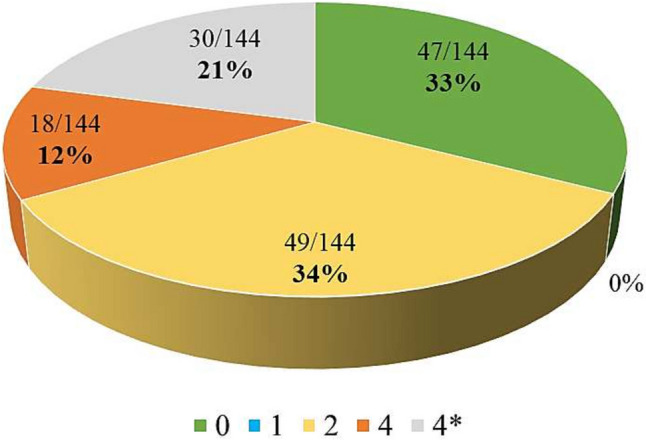
Relative distribution of the quality mapping scores between ORCHESTRA and ISARIC variables according to the ISO/TS 21564:2019 determinant 2 scale

Out of the 144 ISARIC FUP CRF variables (descriptive headers were not considered) for which a mapping score was determined, 96 variables (67%) were either an exact match (47, 33%) or a partial match (49, 34%) to a variable (Fig. 1). We assigned a score of 4/4* to 48 variables. This means, that an abstract map based on rules and guidance could possibly be constructed for them (18, 13%), or they showed no overlap whatsoever (30, 21%).

### Score of 0

Within the variables that were found to show an exact match on semantic domain (score 0), the majority describe common COVID-19 symptoms (21 variables) shown in Table 1.

**Table 1:** Overview of COVID-19 symptom variables that were found to be a match between the ISARIC FUP CRF and ORCHESTRA studies

COVID 19 symptoms
Bleeding
Confusion/ lack of concentration
Diarrhoea
Dizziness/ light-headedness
Fainting/ blackouts
Fatigue
Feeling sick/ vomiting
Headache
Joint pain or swelling
Loss of smell
Loss of taste
Palpitations (heart racing)
Persistent muscle pain
Problems sleeping
Seizures/fits
Shortness of breath/ breathlessness
Skin rash
Stomach / abdominal pain
Tingling feeling/ “pins and needles “
Weight loss
Other Symptoms

An exact match was also found for variables describing demographics (8 variables) as well as 4 variables concerning hospitalization due to COVID-19. Demographics variables included concepts such as date of birth, height and weight, whereas hospitalization contained admission and discharge dates. Furthermore, variables describing COVID-19 treatment (2 variables), such as antivirals and steroids, and COVID-19 vaccination and test date information (1 variable) were also exact matches.

Both the ISARIC and ORCHESTRA studies include breathlessness information which is captured using the Modified Medical Research Council (mMRC) Dyspnea Scale [25].

### Score of 1

As shown in Fig. 1 no variables were given the score of 1. The ORCHESTRA study variables always contained more information than the ISARIC FUP CRF.

### Score of 2

A partial overlap of semantic domain (score 2) was found for 12 variables describing COVID-19 symptoms and also for 4 demographics variables. These symptoms include loss of appetite, muscle weakness and visual and auditory problems whereas demographics information details ethnicity, biological sex at birth and highest educational level. Furthermore, a score of 2 was assigned to 5 variables describing new diagnosis of post-COVID-19 disease, such as stroke, deep vein thrombosis or pulmonary embolism. For these conditions, ORCHESTRA records the severity of the condition and not just its presence.

### Score of 4

A score of 4 was assigned to the “state of health” questionnaires variables defined in the ISARIC FUP CRF capturing responses as evaluated for the date of questionnaire (‘TODAY’). A complex map to ORCHESTRA study variables could possibly be constructed for these variables since they pertain to the same informational domain as those in the 36-Item Short Form Health Survey (SF-36) [26] used in ORCHESTRA studies and could potentially be transformed for sharing purposes. Some variables concerning demographics (e.g. occupation before COVID-19) or reason for hospital re-admission were also scored as 4.

### Score of 4*

No possibility of mapping (denoted by 4*) could be seen for the “state of health” questionnaire variables capturing information for the state pre-COVID-19. In addition, certain COVID-19 symptoms (e.g. erectile dysfunction, changes in menstruation, tremor/shaking) and various occupation-related questions (e.g. concerning sick leave) were not defined as CRF questions in ORCHESTRA studies and assigned a score of 4* as well.

### Scoring by ORCHSTRA study

**Fig. 2:**
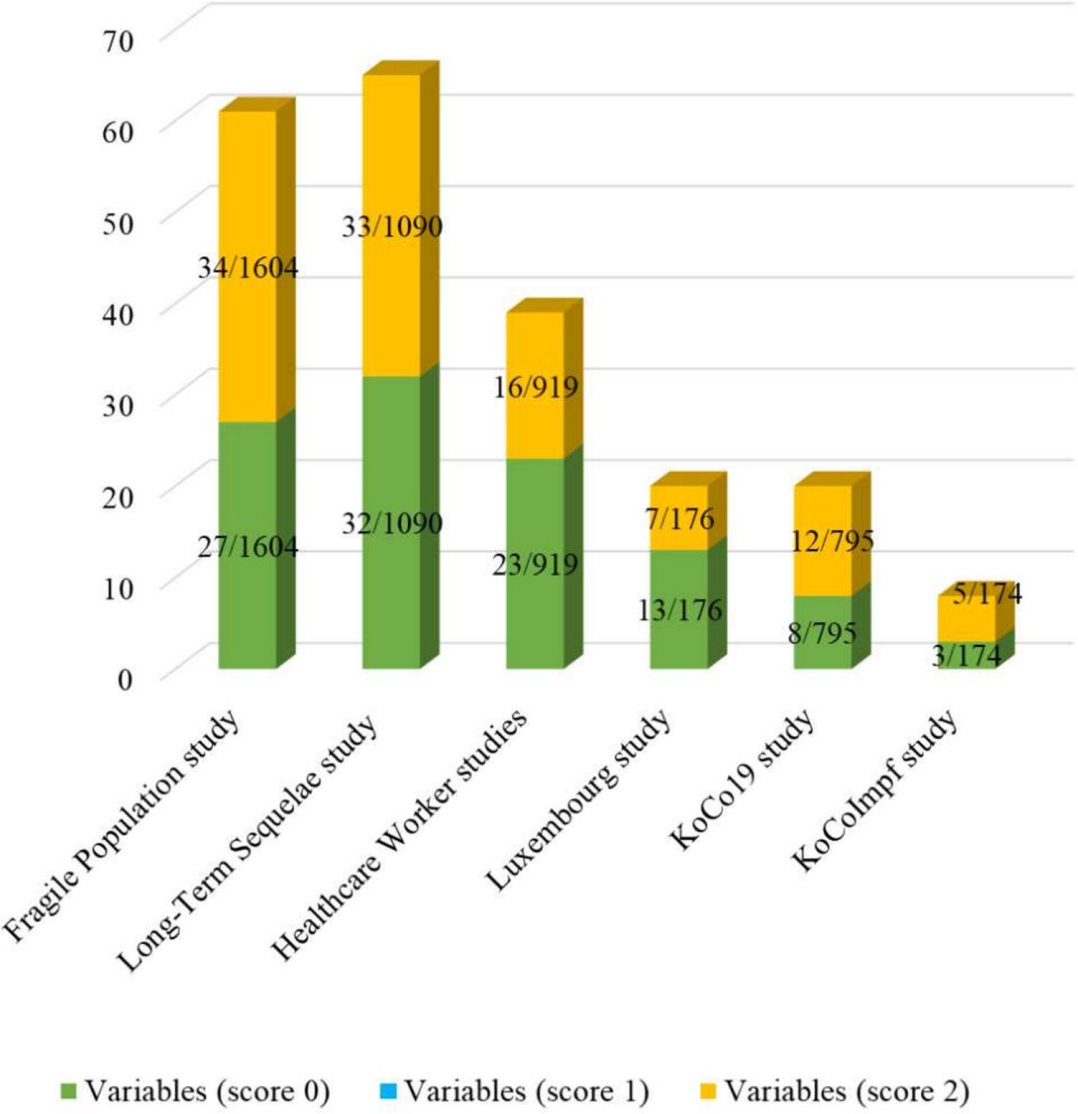
Distribution of assigned mapping scores of 0, 1 or 2 compared to the total number of variables per respective study across the ORCHESTRA studies analyzed in this study

While the ISARIC FUP CRF is just one form, we compared its variables to six studies in ORCHESTRA. An analysis of variables (Fig. 2) with a score of 0, 1 and 2 based on the ORCHESTRA study that includes the respective variable in their CRF, showed the highest number of matching variables or partially matching variables (scores 0,2) for the Long-Term Sequelae (33,32), Fragile population (27,34) and Healthcare Workers (23,16) studies. The high matching score for these three particular studies is consistent with the fact that these CRFs include over 900 variables each across several informational categories. Also, there was coordination between the studies to adopt the same variables wherever possible. KoCoImpf, KoCo19 and the WP3 Luxembourg are much smaller studies in terms of total number of variables and therefore have a limited number of matches with ISARIC FUP CRF.

The overall weighted average score of the map comes to 2.01.

## Discussion

The importance of the ability to merge information from several sources to improve the impact and quality of scientific response has been highlighted throughout the COVID-19 pandemic [27–29]. Initiatives such as the establishment of the European Commission-funded COVID-19 Data Portal [30] and of the Bulletin of the World Health Organization [31], provide examples of tools for sharing clinical study data. A prerequisite for joining data from several studies for analysis is the equivalence of information collected. In order to ensure that data quality does not suffer, this requires the creation of a map between the variables that constitute the datasets that are being considered for combination. In addition, there is oftentimes also a need for time-consuming data transformation.

All the variables in ORCHESTRA have been associated to standard international terminology codes such as those of SNOMED CT, LOINC, ATC, ICD-10 or NCIt that univocally identify the meaning of clinical concepts. Had ISARC variables been associated to international standard codes as was done for ORCHESTRA studies, fully matching variables could have been automatically identified on that basis.

We were able to create a map between the variables that form the ISARIC FUP CRF, and the variables defined as part of six ORCHESTRA studies’ CRFs. Following the ISO/TS 21564:2019 standard for measures of semantic domain, around 67% of the ISARIC FUP CRF variables were assigned a map score of either 0 (exact match), 1 (fully inclusive overlap) or (non-inclusive overlap) and the dataset overall had a score of 2.01. For practical use, the granular results for each score are most relevant in terms of describing the possibility to share clinical study data between research initiatives.

The majority of exactly matching variables between the ISARIC FUP CRF and ORCHESTRA studies were found to center on the description of COVID-19 testing, demographics, hospital admission and symptoms (including breathlessness). Indeed the variables that represent one concept only, like “PCR test result”, tend to be expressed in a similar manner across different studies. Conversely, other variables, more specific to each study’s objective, tend to involve more concepts both in the question and in the possible answer options. For example, the question "Before you got COVID-19 what was your occupation/working status (paid or unpaid)?” investigates the occupational status at a precise time point and even specifies that it refers to both paid and unpaid job. As a result, it is likely that, a question about occupational status, in a different study, would not specify the same details. Additionally, the answer value sets describing possible occupations are not standardized and therefore a perfect match of meaning between the variables is very difficult.

ORCHESTRA variables present a greater informational depth with regards to treatment and underlying conditions. This result was expected since the number of variables included in most ORCHESTRA studies is far greater than those in the ISARIC FUP CRF. Additionally, the ORCHESTRA pool of variables has increasingly been supplemented with new elements stemming from new protocols of study progressively being defined within the European project. The overall ORCHESTRA pool of variables covers several information categories reflecting the variety of studies it includes.

In that context, the ISARIC Tier-1 Ongoing Follow up Survey [32] was also considered for mapping. Since the CRF is less comprehensive than the Freestanding Follow Up Survey, we chose to develop a map for the latter.

Overall, not taking into account contractual and data protection concerns, this analysis shows that the ORCHESTRA project could share data on clinical variables with other studies that have been /are using the ISARIC FUP CRF for data collection. Information concerning symptoms, tests, and admissions could be easily exchanged in such a scenario. Data collected on variables with a map score of 2, which included those providing information about treatment and underlying conditions, would also be a candidate for shared analysis but would require data transformation.

### Limitations

Although we followed the ISO guidelines in order to establish an objective measure of the quality of the semantic mapping performed, subjectivity of assigning map scores by individuals cannot be entirely removed. Re-evaluation of the map would likely result in slightly deviating results if performed by different raters, who perhaps possess a different familiarity with the source or target system variables.

Based on our map, the information that would be the most difficult to exchange would be that contained in questionnaires. This is because questions are typically phrased in a specific manner and embrace more concepts so that even when they cover the same topic extraction of exactly the same information requires a deeper investigation. In this exchange scenario, information collected about enrolled patients’ mental health, education and occupation could only be shared to a limited extent as well and would require further mapping and transformation of the collected data.

### Data sharing possibilities

By September 2021, data for 708,158 patients worldwide had been entered using the ISARIC REDCap® electronic data capture system to the Infectious Diseases Data Observatory (IDDO) platform, using the CCP [33]. While data on the adoption of the ISARIC FUP CRF is not available, based on the figures available for adoption of the ISARIC’s CCP, we can assume a significant number of studies are using it. Thus, there is great potential for data sharing between the ORCHESTRA studies and those other research initiatives. Increasing the sample size (data available) for analysis on a set of variables is highly desirable in order to increase the statistical power of the results (34). An extensive use of international standards and of common variables when designing new CRFs would enable the percentage of matching variables between different studies to reach higher coverage. This way different research questions involving also separate informational categories could efficiently find their answers by combining and analyzing large amounts of data without further transformation.

## Conclusion

This study is the first report of a map created between the ISARIC FUP CRF variables and another research project’s, ORCHESTRA’s study variables, to highlight possibilities of data sharing. Combining data from various studies may provide more power to analytical insights. To enable data aggregation, mapping between two systems is necessary to determine whether further harmonization and transformation are necessary. We believe our study serves as an example of the kind of maps between CRF variables that are needed in order to link many of the ongoing COVID-19 research efforts and pave the road to collaboration and data sharing.

### Supplementary information

Below is the link to the electronic supplementary material.

Supplementary file1 (XLSX 52 KB)

## Data Availability

The datasets generated and/or analyzed during the current study are available in the ORCHESTRA Portal repository, https://cloud.orchestra-cohort.eu/s/H5kkiAA55spHXpf.

## Abbreviations

CRF: Case Report Form
ISARIC: International Severe Acute Respiratory and Emerging Infection Consortium
ISARIC FUP: ISARIC Freestanding Follow Up Survey
WHO: World Health Organization
CCP: Clinical Characterization Protocol
ISO/TS: International Organization for Standardization/ Technical Specification
mMRC Dyspnea Scale: Modified Medical Research Council Dyspnea Scale
SF-36: 36-Item Short Form Health Survey
IDDO: Infectious Diseases Data Observatory

## References

[1] Nishimura, K. et al. Comparison between electronic and paper versions of patient-reported outcome measures in subjects with chronic obstructive pulmonary disease: an observational study with a cross-over administration. BMJ Open 9, e032767 (2019). 10.1136/bmjopen-2019-032767 PMC6937099 31857313

[2] Zimmermann, P., Pittet, L. F. & Curtis, N. The Challenge of Studying Long COVID: An Updated Review. Pediatr Infect Dis J 41, 424–426 (2022). 10.1097/INF.0000000000003502 PMC8997013 35213866

[3] Angelis A, Suarez Alonso C, Kyriopoulos I, Mossialos E (2022). Funding Sources of Therapeutic and Vaccine Clinical Trials for COVID-19 vs Non–COVID-19 Indications, 2020–2021. JAMA Netw. Open.

[4] A Azzini, A. M. et al. How European Research Projects Can Support Vaccination Strategies: The Case of the ORCHESTRA Project for SARS-CoV-2. Vaccines (Basel) 11, 1361 (2023). 10.3390/vaccines11081361 PMC10459328 37631929

[5] Fleischmann R, Decker AM, Kraft A, Mai K, Schmidt S (2017). Mobile electronic versus paper case report forms in clinical trials: a randomized controlled trial. BMC Med Res Methodol.

[6] Bushnell DM, Martin ML, Parasuraman B (2003). Electronic versus paper questionnaires: a further comparison in persons with asthma. J Asthma.

[7] Nishimura K, Kusunose M, Sanda R, Tsuji Y, Hasegawa Y, Oga T (2019). Comparison between electronic and paper versions of patient-reported outcome measures in subjects with chronic obstructive pulmonary disease: an observational study with a cross-over administration. BMJ Open.

[8] Le Jeannic A, Quelen C, Alberti C, Durand-Zaleski I, CompaRec Investigators (2014). Comparison of two data collection processes in clinical studies: electronic and paper case report forms. BMC Med Res Methodol.

[9] Ray, Sumantra (Shumone), and others (eds), 'Data capture tools: case report form (CRF)', in Sumantra Ray, and others (eds), Oxford Handbook of Clinical and Healthcare Research, 1, Oxford Medical Handbooks (Oxford, 2016; online edn, Oxford Academic, 1 Mar. 2016), 10.1093/med/9780199608478.003.0016, accessed 8 Nov. 2023.

[10] ISARIC Clinical Characterization Group et al. ISARIC-COVID-19 dataset: A Prospective, Standardized, Global Dataset of Patients Hospitalized with COVID-19. Sci Data 9, 454 (2022). 10.1038/s41597-022-01534-9 PMC9339000 35908040

[11] ISARIC Clinical Characterisation Group. The value of open-source clinical science in pandemic response: lessons from ISARIC. Lancet Infect Dis 21, 1623–1624 (2021). 10.1016/S1473-3099(21)00565-X PMC8489876 34619109

[12] Sass J, Bartschke A, Lehne M, Essenwanger A, Rinaldi E, Rudolph S (2020). The German Corona Consensus Dataset (GECCO): a standardized dataset for COVID-19 research in university medicine and beyond. BMC Med. Inf. Decis. Making.

[13] Akbarialiabad H, Taghrir MH, Abdollahi A, Ghahramani N, Kumar M, Paydar S (2021). Long COVID, a comprehensive systematic scoping review. Infection.

[14] Rinaldi E, Stellmach C, Rajkumar NMR, Caroccia N, Dellacasa C, Giannella M (2022). Harmonization and standardization of data for a pan-European cohort on SARS- CoV-2 pandemic. NPJ Digit Med.

[15] Arvanitis, T. N. Semantic interoperability in healthcare. Stud Health Technol Inform 202, 5–8 (2014). 25000001

[16] A. Tolk, "Interoperability, Composability, and Their Implications for Distributed Simulation: Towards Mathematical Foundations of Simulation Interoperability," 2013 IEEE/ACM 17th International Symposium on Distributed Simulation and Real Time Applications, Delft, Netherlands, 2013, pp. 3-9, 10.1109/DS-RT.2013.8.

[17] Hallock, H. et al. Federated Networks for Distributed Analysis of Health Data. Front Public Health 9, 712569 (2021). 10.3389/fpubh.2021.712569 PMC8514765 34660512

[18] Rolland B, Reid S, Stelling D, Warnick G, Thornquist M, Feng Z (2015). Toward Rigorous Data Harmonization in Cancer Epidemiology Research: One Approach. Am J Epidemiol..

[19] International Organization for Standardization. (2019). Health Informatics — Terminology resource map quality measures (MapQual) (ISO Standard No. 21564:2019). https://www.iso.org/standard/71088.html

[20] ISARIC. (2021). Tier-1-Ongoing-Follow-up-Survey. https://isaric.org/wp-content/uploads/2021/03/Initial-Freestanding-survey.pdf

[21] Gentilotti, E. et al. Clinical phenotypes and quality of life to define post-COVID-19 syndrome: a cluster analysis of the multinational, prospective ORCHESTRA cohort. eClinicalMedicine 62, (2023). 10.1016/j.eclinm.2023.102107 PMC10466236 37654668

[22] Giannella, M. et al. Using machine learning to predict antibody response to SARS-CoV-2 vaccination in solid organ transplant recipients: the multicentre ORCHESTRA cohort. Clin Microbiol Infect (2023) 10.1016/j.cmi.2023.04.027. 10.1016/j.cmi.2023.04.027 PMC10212001 37150358

[23] Radon K, Saathoff E, Pritsch M, GuggenbühlNoller JM, Kroidl I, Olbrich L (2020). Protocol of a population-based prospective COVID-19 cohort study Munich, Germany (KoCo19). BMC Public Health.

[24] Reinkemeyer, C. et al. The Prospective COVID-19 Post-Immunization Serological Cohort in Munich (KoCo-Impf): Risk Factors and Determinants of Immune Response in Healthcare Workers. Viruses 15, 1574 (2023). 10.3390/v15071574 PMC10383736 37515259

[25] Casanova C, Marin JM, Martinez-Gonzalez C, de Lucas-Ramos P, Mir-Viladrich I, Cosio B (2015). Differential Effect of Modified Medical Research Council Dyspnea, COPD Assessment Test, and Clinical COPD Questionnaire for Symptoms Evaluation Within the New GOLD Staging and Mortality in COPD. CHEST.

[26] Lins L, Carvalho FM (2016). SF-36 total score as a single measure of health-related quality of life: Scoping review. SAGE Open Med..

[27] Dron L, Dillman A, Zoratti MJ, Haggstrom J, Mills EJ, Park JJH (2021). Clinical Trial Data Sharing for COVID-19–Related Research. J. Med. Internet Res..

[28] Li R, von Isenburg M, Levenstein M, Neumann S, Wood J, Sim I (2021). COVID-19 trials: declarations of data sharing intentions at trial registration and at publication. Trials.

[29] Fegan G, Cheah PY (2021). Solutions to COVID-19 data sharing. Lancet Digit Health.

[30] Harrison PW, Lopez R, Rahman N, Allen SG, Aslam R, Buso N (2021). The COVID-19 Data Portal: accelerating SARS-CoV-2 and COVID-19 research through rapid open access data sharing. Nucleic Acids Res..

[31] Moorthy V, HenaoRestrepo AM, Preziosi MP, Swaminathan S (2020). Data sharing for novel coronavirus (COVID-19). Bull World Health Organ..

[32] ISARIC. (2021). Tier-1-Ongoing-Follow-up-Survey. https://isaric.org/wp-content/uploads/2020/12/Tier-1-Ongoing-Follow-up-Survey.pdf

[33] Garcia-Gallo E, Merson L, Kennon K, Kelly S, Citarella BW, Fryer DV (2022). ISARIC-COVID-19 dataset: A Prospective, Standardized, Global Dataset of Patients Hospitalized with COVID-19. Sci Data..

[34] Serdar CC, Cihan M, Yücel D, Serdar MA (2021). Sample size, power and effect size revisited: simplified and practical approaches in pre-clinical, clinical and laboratory studies. Biochem Med (Zagreb)..

